# Hyperbaric oxygen therapy vs. pharmacological intervention in adults with fibromyalgia related to childhood sexual abuse: prospective, randomized clinical trial

**DOI:** 10.1038/s41598-024-62161-5

**Published:** 2024-05-21

**Authors:** Rahav Boussi-Gross, Merav Catalogna, Erez Lang, Zipora Shamai, Jacob N. Ablin, Valerie Aloush, Keren Doenyas-Barak, Mordechai Lorberboym, Rachel Lev-Wiesel, Shai Efrati

**Affiliations:** 1 Sagol Center for Hyperbaric Medicine and Research, Shamir (Assaf Harofeh) Medical Center, Zerifin, Israel; 2 School of Medicine, Tel-Aviv University, Tel-Aviv, Israel; 3 Sagol School of Neuroscience, Tel-Aviv University, Tel-Aviv, Israel; 4 Nuclear Medicine Institute, Shamir (Assaf Harofeh) Medical Center, Zerifin, Israel; 5 The Emili Sagol CAT Research Center, Graduate School of Creative Arts Therapies, University of Haifa, Haifa, Israel; 6 Tel Aviv Sourasky Medical Center, Tel-Aviv, Israel

**Keywords:** Fibromyalgia, Outcomes research

## Abstract

Fibromyalgia syndrome (FMS) is a chronic pain syndrome characterized by disruptions in pain processing within the central nervous system. It exhibits a high prevalence among patients with a history of traumatic experiences, notably childhood sexual abuse (CSA). This study compared the efficacy of hyperbaric oxygen therapy (HBOT) to the current pharmacological standard of care for individuals suffering from CSA-related FMS. Forty-eight participants diagnosed with FMS and a history of CSA were randomly assigned to either the HBOT group (60 sessions of 100% oxygen at 2 ATA for 90 min, with air breaks every 5 min) or the medication (MED) group (FDA-approved medications, Pregabalin and Duloxetine). The primary endpoint was the Fibromyalgia impact questionnaire (FIQ) score, while secondary endpoints encompassed emotional status and daily functioning questionnaires, as well as pain thresholds and conditioned pain modulation tests. Brain activity was evaluated through single photon emission computed tomography (SPECT). Results revealed a significant group-by-time interaction for the FIQ score favoring HBOT over MED (p < 0.001), with a large effect size (Cohen's d = − 1.27). Similar findings were observed in emotional symptoms and functional measures. SPECT imaging demonstrated an increase in activity in pre-frontal and temporal brain areas, which correlated with symptoms improvement. In conclusion, HBOT exhibited superior benefits over medications in terms of physical, functional, and emotional improvements among FMS patients with a history of CSA. This associated with increased activity in pre-frontal and temporal brain areas, highlighting the neuroplasticity effect of HBOT.

## Introduction

Fibromyalgia syndrome (FMS) is a debilitating condition characterized by chronic widespread pain, along with symptoms of fatigue, disrupted sleep, and cognitive dysfunction^1,2^. In recent years, significant progress has been made in understanding the pathogenesis of FMS, with a growing consensus that it represents a prototype of central sensitization—a condition characterized by disruptions in the transmission and processing of pain within the central nervous system (CNS)^3,4^. While the etiology of FMS remains incompletely understood, it is increasingly recognized that a complex interplay between genetic predisposition and exposure to various triggers underlies many cases. Among these triggers, traumatic experiences, including childhood sexual, emotional, or physical abuse, have been suggested as predisposing factors in a substantial number of FMS patients^5,6^.

Childhood trauma, such as childhood sexual abuse (CSA), can have profound and enduring effects on an individual's neurobiology. Long-term exposure to trauma during critical and sensitive developmental periods can lead to lasting alterations in brain structure and function, as well as disruptions in brain's reaction to stress and painful stimuli^7–13^. These persistent neurobiological changes can manifest as physical pain, emotional and functional dysfunctions, and other health risks in adulthood, even many years after the traumatic experiences^7–13^.

Current treatment strategies for FMS rely on multidisciplinary interventions, with a modest role for pharmacological agents^14–16^. The treatment guidelines for FMS recommend medications that modulate the serotonin and noradrenaline reuptake inhibitor (SNRI) antidepressants or medications that modulate the gamma-aminobutyric acid (GABA) neurotransmitter in the brain and CNS, coupled with regular physical exercise^14–16^. However, despite the optimal implementation of these methods, success rates remain modest, and FMS continues to pose an unmet clinical challenge.

In recent years, a growing body of evidence has emerged regarding the neuroplasticity-inducing effects of new hyperbaric oxygen therapy (HBOT) protocols^17–24^. HBOT involves the application of elevated atmospheric pressure combined with increased oxygen levels, enhancing the diffusion of oxygen to poorly perfused tissues. The new HBOT protocol, utilizing the hyperoxic–hypoxic paradox (HHP), is among the first therapeutic interventions in clinical use today specifically designed to promote the regeneration of damaged brain tissue^17,18,22^. HHP represents a novel paradigm aimed at bolstering endogenous repair mechanisms while providing an optimal microenvironment. These effects encompass the stimulation, migration, and differentiation of stem cells, mitochondrial proliferation/biogenesis, mitochondrial transfer, and angiogenesis^17^.

The beneficial effects of HBOT have been demonstrated in individuals with persistent impaired brain functions, even years after an acute insult, across various populations such as post-stroke, post-concussion, and post-COVID patients^21,22,25–30^. HBOT protocols have also shown promise in inducing neuroplasticity and improving clinical symptoms in veterans with treatment-resistant military-related post-traumatic stress disorder (PTSD)^31–33^. Furthermore, HBOT has emerged as a promising modality for a subgroup of FMS patients who developed the syndrome following severe emotional stress, including childhood abuse^20,34^. However, it remains unexplored whether the effects of HBOT surpass those of the current recommended pharmacological treatments for patients suffering from FMS related to CSA.

In the present study, we sought to evaluate the therapeutic efficacy of HBOT in FMS patients triggered by past traumatic experiences of CSA and to compare it with the standard approved pharmacological treatment. Our objective was to assess the clinical utility of HBOT vs. pharmacotherapy in this specific subgroup of FMS patients while investigating the neuroplasticity effects of the intervention through metabolic brain imaging.

## Results

### Participants’ characteristics and randomization

**Figure 1:**
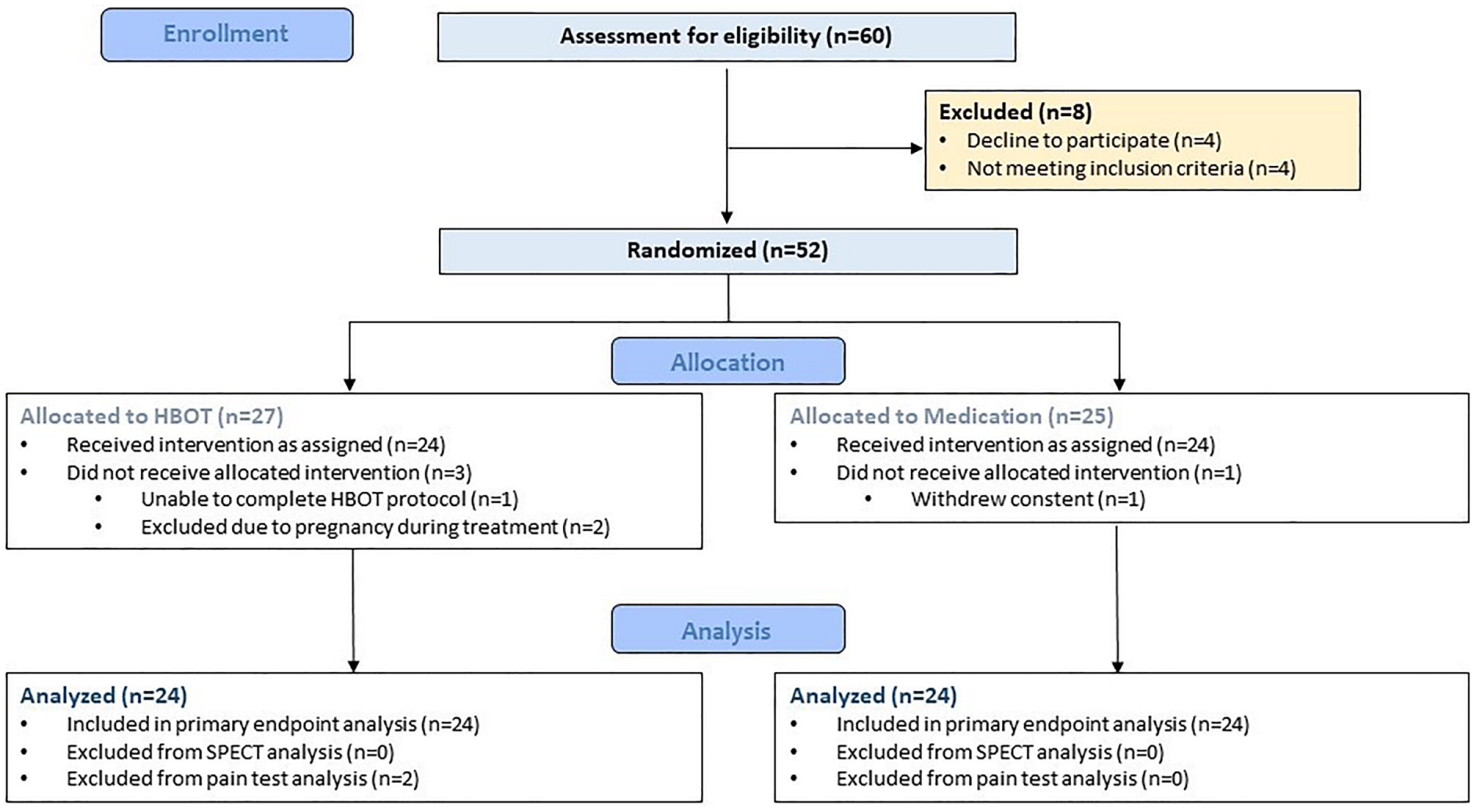
Study flowchart. *HBOT* Hyperbaric oxygen therapy, *SPECT* Single-photon emission computed tomography.

Sixty participants signed informed consent in this study. Eight were excluded or declined participation prior to randomization. Therefore, 52 participants were randomized to two active arms, 27 to HBOT group, and 25 to medication (MED) group. In HBOT, one participant was unable to complete the treatment protocol due to inability to compare pressures, two participants were excluded after several treatment sessions due to pregnancy. In Med, one participant withdrew her consent after allocation. Eventually, data of 48 participants (24 in each active arm) was included in final analysis. Flowchart of all participants and inclusion in primary and secondary end points is presented in Fig. 1. Demographics and baseline characteristics of participants are detailed in Table 1, with no significant differences between the two groups, except for criterion of age of abuse (first memory).

**Table 1:** Baseline characteristics.

	HBOT group	Medications group	p-value
N	24	24	
Age	32.8 ± 6.3	34.0 ± 5.6	0.49
Years of education	14.3 ± 2.6	15.0 ± 3.3	0.44
*Marital status*			
Single	13 (54.2)	8 (33.3)	0.24
Married	7 (29.2)	14 (58.3)	0.08
Divorced	2 (8.3)	1 (4.2)	1.00
Live with a partner	2 (8.3)	1 (4.2)	1.00
Number of children	0.8 ± 1.4	1.0 ± 1.5	0.61
Employment status (employed)	9 (37.5)	12 (50.0)	0.56
*Childhood trauma measures (CTQ)*			
Emotional abuse	16.7 ± 6.1	17.8 ± 5.3	0.50
Physical abuse	11.2 ± 5.9	9.7 ± 5.5	0.37
Sexual abuse	15.8 ± 6.6	17.5 ± 5.7	0.35
Emotional neglect	16.2 ± 5.8	18.8 ± 4.5	0.10
Physical neglect	10.8 ± 4.3	12.0 ± 4.5	0.39
*Fibromyalgia measures*			
Wide spread pain index (WPI)	13.9 ± 4.1	13.1 ± 3.9	0.50
Symptom severity score (SSS)	10.3 ± 1.6	10.5 ± 1.1	0.68
Past psychotherapy treatment (years)	4.4 ± 3.3	5.2 ± 4.4	0.51
Past hospitalizations	6 (25.0)	6 (25.0)	1.00
Previous suicidal attempts	3 (12.5)	4 (16.7)	1.00
*Measures of childhood assault*			
*Identity of offender*			
Incest (parent of sibling)	14 (58.3)	12 (50.0)	0.77
Other	10 (41.7)	12 (50.0)	1.00
*Frequency of abuse*			
Unknown	1 (4.2)	2 (8.3)	1.00
One time	5 (20.8)	3 (12.5)	0.69
Multiple	4 (16.7)	9 (37.5)	0.50
Continuous (> year)	14 (58.3)	10 (41.7)	0.42
Age of abuse (first memory)	9.5 ± 4.8	6.7 ± 3.8	0.03
*Number of offenders*			
Single	19 (79.2)	21 (87.5)	0.70
Multiple	5 (20.8)	3 (12.5)	1.00
*Related background medical conditions*			
Asthma (childhood)	3 (12.5)	0 (0.0)	0.23
Hypothyroidism	6 (25.0)	1 (4.2)	0.09
IBS	3 (12.5)	2 (8.3)	1.00
Eating disorders	1 (4.2)	3 (12.5)	0.60
Endometriosis	2 (8.3)	3 (12.5)	1.00
PCOS/ovarian cyst	1 (4.2)	3 (12.5)	0.60
ADHD	4 (16.7)	6 (25.0)	0.72
BMI (kg/m^2^)	40.1 ± 9.9	36.1 ± 7.3	0.12
*Disease-related chronic medications*			
Cannabis	7 (29.2)	8 (33.3)	1.00
Benzodiazepines	4 (16.7)	3 (12.5)	1.00
Antidepressants	14 (58.3)	9 (37.5)	0.24
Antipsychotics	4 (16.7)	3 (12.5)	1.00
Anticonvulsants	4 (16.7)	2 (8.3)	0.66
Levothyroxine	5 (20.8)	1 (4.2)	0.18
Attention disorder treatment	2 (8.3)	3 (12.5)	1.00

Data presented as n (%); continuous data, mean ± SD. *HBOT* Hyperbaric oxygen therapy, *IBS* irritable bowel syndrome, *PCOS* polycystic ovary syndrome, *ADHD* attention-deficit/hyperactivity disorder, *BMI* body mass index.

### Primary outcome

**Figure 2:**
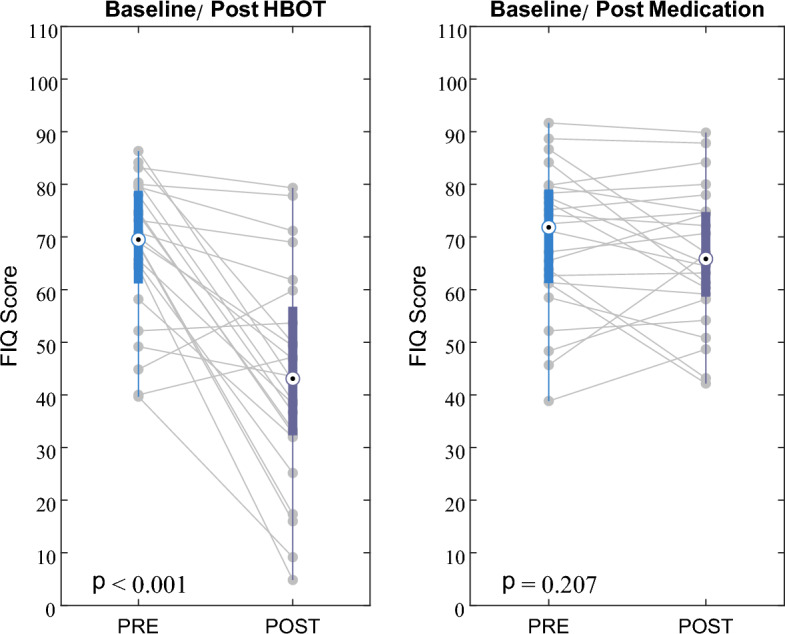
Fibromyalgia impact questionnaire (FIQ) score changes in HBOT and medications groups. Significant Group-by-Time interaction effect, with large effect size (p < 0.001, Cohen’s d = − 1.27). Data shown in boxplots, the central mark indicates the median, and the bottom and top edges of the box indicate the 25th and 75th percentiles, respectively.

A significant group-by-time interaction was found in fibromyalgia impact questionnaire (FIQ) total score, with large effect size (d = − 1.27, p < 0.001; Fig. 2). Significant interaction was also found in all subscales of this measure (Table 2).

**Table 2:** Questionnaire analysis.

	HBOT Group				MED Group						ANOVA**	
	PRE	POST	Change	p-value	PRE	POST	Change	p-value	p-value Baseline	Effect size*	F	p-value
N	24				24							
Wide spread pain index (WPI)	13.9 ± 4.1	9.4 ± 6.0	− 4.5 ± 5.9	0.001	13.1 ± 3.9	13.4 ± 2.9	0.3 ± 3.9	0.717	0.503	− 0.971	11.322	**0.002**
Symptom severity score (SSS)	10.3 ± 1.6	8.0 ± 2.5	− 2.3 ± 2.8	0.001	10.5 ± 1.1	10.4 ± 1.2	− 0.1 ± 1.4	0.671	0.681	− 0.988	11.706	**0.001**
Fibromyalgia impact questionnaire-revised (FIQ-R)												
Total FIQ-R	67.5 ± 13.4	43.2 ± 20.0	− 24.3 ± 20.9	< 0.001	69.2 ± 13.6	66.3 ± 12.8	− 2.9 ± 11.1	0.207	0.665	− 1.277	19.562	** < 0.001**
Function	18.0 ± 5.9	11.7 ± 6.2	− 6.4 ± 6.4	< 0.001	19.6 ± 5.9	19.1 ± 5.6	− 0.5 ± 4.7	0.631	0.386	− 1.050	13.239	**0.001**
Overall impact	13.8 ± 4.5	6.7 ± 5.7	− 7.1 ± 6.9	< 0.001	12.8 ± 4.3	11.5 ± 4.0	− 1.2 ± 3.9	0.129	0.446	− 1.043	13.058	**0.001**
Symptoms	35.6 ± 6.5	24.8 ± 10.4	− 10.8 ± 11.4	< 0.001	36.9 ± 5.7	35.6 ± 5.6	− 1.2 ± 4.8	0.231	0.506	− 1.100	14.525	** < 0.001**
Brief symptoms inventory (BSI)												
Total BSI	45.3 ± 13.4	32.1 ± 19.1	− 13.2 ± 21.6	0.006	46.2 ± 9.9	44.7 ± 9.2	− 1.5 ± 11.4	0.514	0.793	− 0.677	5.499	0.023
PTSD symptom scale (PSS)												
PTSD severity	35.2 ± 9.1	25.4 ± 12.8	− 9.8 ± 12.6	0.001	36.0 ± 7.1	35.1 ± 8.9	− 0.9 ± 8.7	0.610	0.743	− 0.824	8.150	0.006
BECK depression inventory (BDI)												
	33.8 ± 11.2	24.2 ± 15.3	− 9.6 ± 15.4	0.006	35.3 ± 12.0	35.6 ± 9.3	0.3 ± 10.9	0.883	0.662	− 0.744	6.638	0.013
Medical somatic dissociation questionnaire (MSDQ)												
Total MSDQ	2.1 ± 0.6	1.5 ± 0.7	− 0.6 ± 0.8	0.001	2.2 ± 0.7	2.2 ± 0.7	0.0 ± 0.4	0.704	0.780	− 1.014	12.336	**0.001**
RAND health status survey short form-36												
Physical component summary	23.8 ± 12.4	47.9 ± 21.5	24.1 ± 17.9	< 0.001	26.6 ± 14.0	27.5 ± 12.6	0.9 ± 13.6	0.7521	0.475	1.464	25.730	** < 0.001**
Mental component summary	24.0 ± 13.1	48.3 ± 21.5	24.3 ± 20.8	< 0.001	23.6 ± 10.5	28.2 ± 14.5	4.6 ± 17.1	0.2026	0.913	1.039	12.966	**0.001**

Data are presented as mean ± SD; Bold, significant after Bonferroni correction; *Cohen's d net effect size; **group-by-time interaction. *HBOT* Hyperbaric oxygen therapy, *MED* medications, *ANOVA* analysis of variance, *PTSD* post traumatic stress disorder

### Secondary outcomes

**Figure 3:**
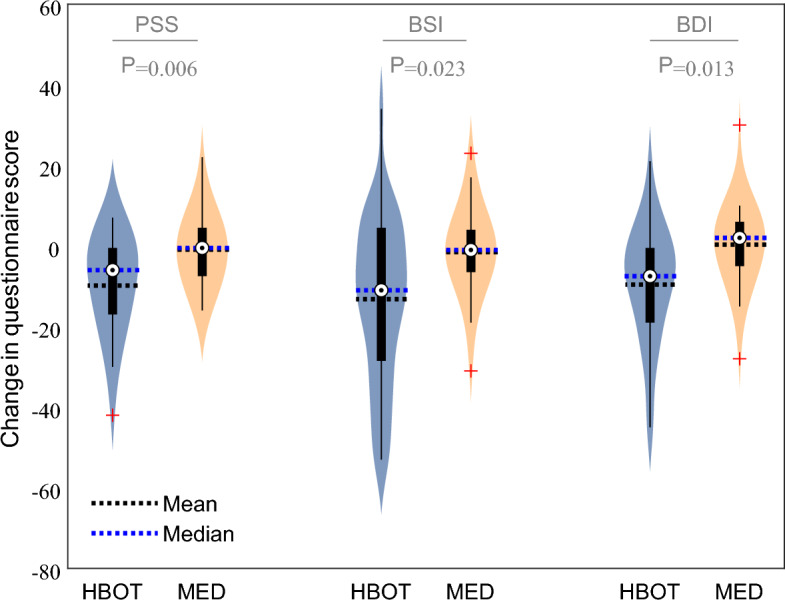
Changes in questionnaires scores in HBOT and Med groups. p-values represent the significance of group-by-time interactions for each questionnaire. Data are presented in violin and boxplots, the bottom and top edges of the box indicate the 25th and 75th percentiles, respectively. Red symbols indicate outliers. *PSS* PTSD symptoms scale, *BSI* Brief symptom inventory, *BDI* Beck depression inventory, *HBOT* Hyperbaric oxygen therapy, *MED* Medications.

Main results of questionnaires analysis are summarized in Table 2 and Fig. 3 (extended results of all questionnaires’ sub-measures in Supplementary Table S1). At baseline, no significant difference was found between the groups in the different domains. At baseline, all participants demonstrated positive FMS diagnosis criteria (calculated from wide spread pain index—WPI and symptoms severity score—SSS measures^35^). Following treatments, 7 participants in the HBOT group (29%) no longer met FMS criteria, compared to none in the MED group.

Emotional symptoms demonstrated significant improvement after HBOT compared to MED intervention: Significant group-by-time interaction was found in PTSD severity according to PTSD symptom scale (PSS) scores with large effect size (d = − 0.82, p < 0.01); In medical somatic dissociation questionnaire (MSDQ) total score with large effect size (d = − 1.01, p < 0.005); In brief symptoms inventory (BSI) total score with medium effect size (d = − 0.67, p < 0.05), and in Beck depression inventory (BDI) total score with medium effect size (d = − 0.74, p < 0.05). All sub-scales interactions of these measures were also found significant (Supplementary Table S1).

RAND health status survey short form 36 (SF36) questionnaire demonstrated significant improvement in both physical component score (p < 0.001) and mental component score (p < 0.005), with large Cohen’s d effect sizes (d = 1.46, 1.03 respectively). All sub-scales interactions of SF36 were also found significant (Supplementary Table S1).

### Pain test

Two participants from the HBOT group were excluded from pain test analysis due to technical reasons. In the HBOT group, PPT increased in 70.8 ± 90.6 kPa, compared to increase of 20.1 ± 85.7 kPa in the MED group, group-by-time interaction demonstrated marginal significance with medium effect size (d = 0.57, p = 0.05). PPT in the immersed condition has demonstrated increase in similar rate in the HBOT condition 73.6 ± 121.4 kPa and in smaller rate in the MED intervention, of 4.4 ± 121.2 kPa, with marginal significance of group-by-time interaction and medium effect size (d = 0.57, p = 0.06). Group-by-time interaction of conditioned pain modulation (CPM) score was not significant (p = 0.56). Detailed data of pain tests of both groups are presented in Supplementary Table S2.

### Brain activity imaging

**Figure 4:**
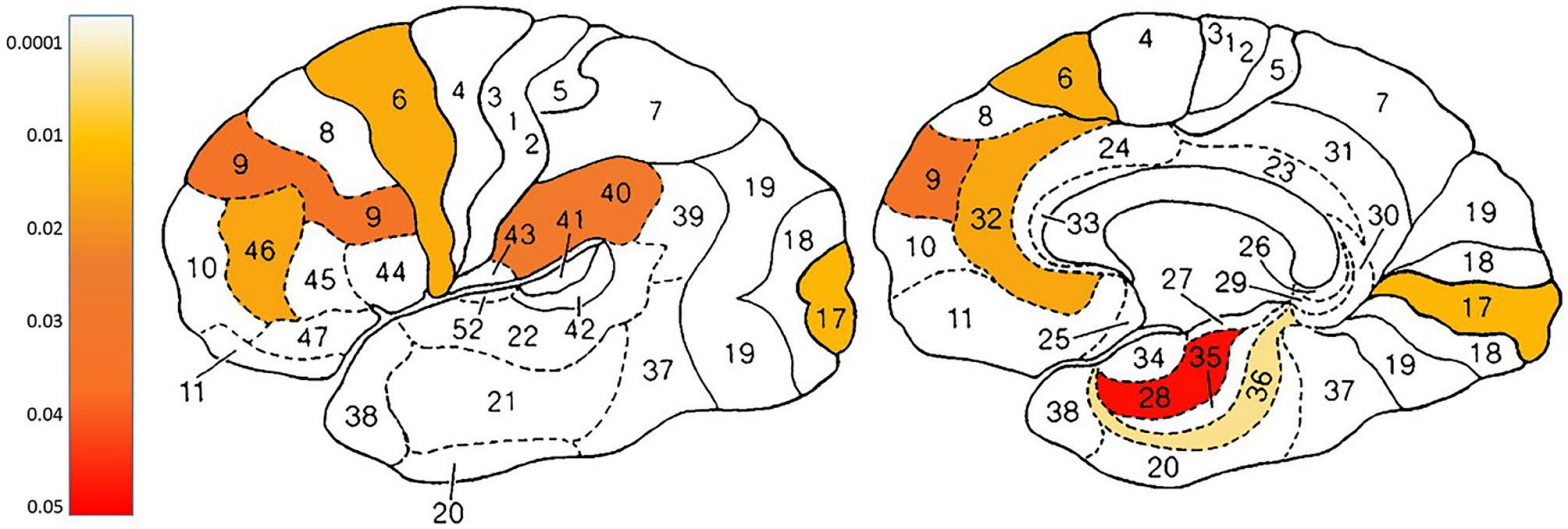
Single-photon emission computed tomography (SPECT) analysis. Increased post treatment activation in Brodmann areas in hyperbaric oxygen therapy (HBOT) group compared to medications group. p-values represent the group-by-time interaction of SPECT data.

**Figure 5:**
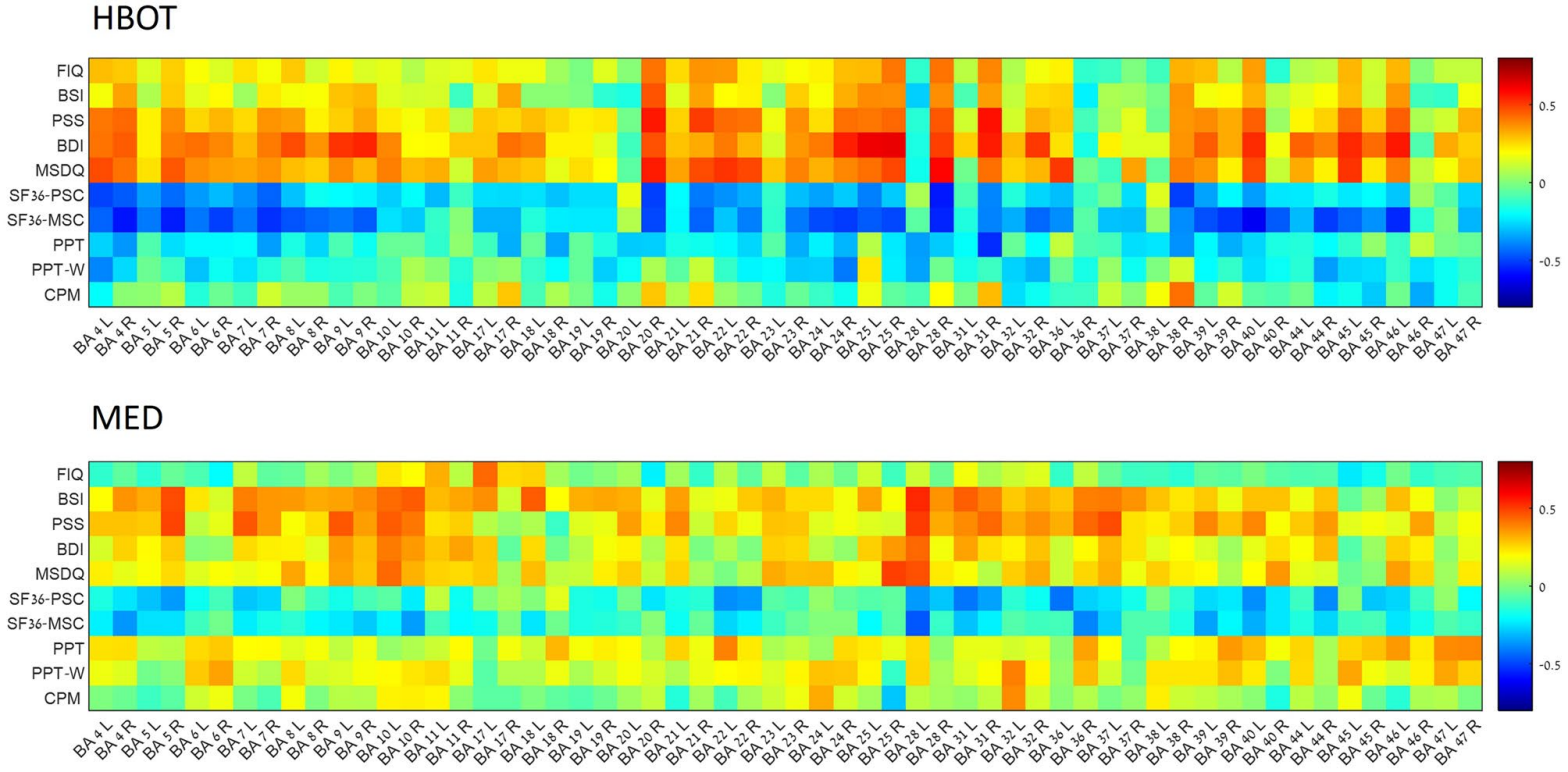
Correlations heatmap. Pearson correlation coefficient heatmap representing post intervention changes in Brodmann areas (BA) activation, pain test and questionnaire scores in Hyperbaric oxygen therapy (HBOT) and medications (MED) groups. *FIQ* Fibromyalgia impact questionnaire, *BSI* Brief symptoms inventory, *PSS* PTSD symptoms scale, *BDI* Beck depression inventory, *MSDQ* Medical somatic dissociation questionnaire, *SF36-PSC* RAND health status survey short form-36—physical component summary, *SF36-MSC* RAND health status survey short form-36—mental component summary, *PPT* Pressure pain threshold, *PPT-W* Pressure pain threshold in water, *CPM* Conditioned pain modulation.

Results of SPECT analysis are presented in Table 3 and Fig. 4. Four Brodmann areas (BA) were found with a significant increase in activity for the HBOT group, and a significant group-by-time interaction: left BA6 (p = 0.01), left BA36 (p = 0.001), right BA40 (p = 0.02), left BA46 (p = 0.01). Two BAs were found with a significant decrease in the MED group and a significant group-by-time interaction: right BA17 (p = 0.01) and left BA32 (p = 0.01). Other BAs with group-by-time effects were right BA9 (p = 0.03), and left BA28 (p = 0.04). however, none were significant after FDR correction. Analysis of all BAs is presented in Supplementary Table S3. Heatmap presentations of correlations between deltas of brain areas’ SPECT activation and deltas of questionnaires and pain test data, for both HBOT and MED groups, are presented in Fig. 5).

**Table 3:** SPECT data. Significant group-by-time interactions by Brodmann area.

	HBOT group p-value	MED group p-value	Baseline comparison p-value	Net effect size*	F	ANOVA p-value**
N	24	24				
BA 6 LEFT	**0.006**	0.545	0.084	0.73	6.42	**0.015**
BA 9 RIGHT	0.095	0.200	0.937	0.63	4.75	**0.035**
BA 17 RIGHT	0.582	**< 0.001**	0.092	0.75	6.80	**0.012**
BA 28 LEFT	0.063	0.324	0.662	0.58	4.09	**0.049**
BA 32 LEFT	0.161	**0.048**	0.644	0.72	6.29	**0.016**
BA 36 LEFT	**0.004**	0.249	0.144	0.95	10.80	**0.002**
BA 40 RIGHT	**0.016**	0.531	0.189	0.65	5.11	**0.029**
BA 46 LEFT	**0.033**	0.238	0.444	0.73	6.34	**0.015**

*Cohen's d net effect size, **Group-by-time interaction. *SPECT* Single-photon emission computed tomography, *HBOT* Hyperbaric oxygen therapy, *MED* Medications, *ANOVA* Analysis of variance, *BA* Brodmann area.

Significant values are in bold.

### Side effects and treatment tolerance

In the MED group, 90% were treated with Pregabalin and 10% with Duloxetine, according to clinical judgement. The most common side effects in the MED group were increased fatigue (37%), increased pain (29%), increased emotional distress (25%) and digestion/stomach pain (20%). Other side effects included weakness and faint, weight gain, headache, limb numbness and sleep disturbance. Some of the participants experienced mood improvement (8%). Fourteen participants (58%) did not tolerate the treatment due to the side effects and decided to stop medication administration prior to the end of 12 weeks’ period (mean time for medication administration in MED group: 5.8 ± 4.6 weeks).

In the HBOT group the most common side effect was mild middle ear barotrauma (29%) that resolved spontaneously after taking a few days off treatment. Other side effects were emotional distress (12.5%) along parts of the treatment course and surfacing of inaccessible memories from childhood (8%). One participant experienced temporary blurred vision during the treatments. One participant discontinued treatments prior to completion (treatment 53/60) due to extreme emotional distress, and per her request to discontinue. Another participant discontinued the treatments after six sessions following her request, due to unresolved sinusoidal pain during pressure elevation. Accordingly, 92% completed the 60 sessions of HBOT as planned.

## Discussion

In this prospective, randomized, two-active-arms trial, we aimed to investigate the effects of HBOT compared to the current standard medication treatment in a population of young women with FMS related to a history of CSA. Our study demonstrated that HBOT induced significant improvements in both physical and emotional symptoms, as well as daily functioning, surpassing the effects of pharmacological treatment. Furthermore, these clinical benefits were in concordance with the neuroplasticity effects observed through brain SPECT evaluation.

A growing body of evidence suggests that the pathophysiological changes resulting from traumatic events can lead to enduring alterations in brain structure and function. These alterations encompass abnormalities in the prefrontal cortex (PFC), the anterior cingulate cortex and the limbic system, as well as impaired connectivity—both functional and structural—between the amygdala, hippocampus, and frontal lobes^33,36,37^. CSA, characterized by traumatic exposures during maturation, can negatively impact brain development, connectivity, and functionality, making it a risk factor for long-term psychopathology^38^. Remarkably, 20–50% of FMS patients have been reported to have a history of sexual abuse, with a relative risk of 2.5–3.1 times higher than that of control groups^6^.

In our study, brain perfusion activity was assessed using SPECT imaging. Post-treatment SPECT scans were performed more than one week after the last HBOT session to mitigate potential intermittent effects of oxygen exposure on the brain. Significant changes in brain perfusion following HBOT, compared to medication, were primarily observed in prefrontal and temporal regions (Fig. 4) and in clusters of brain areas significantly correlated with symptom improvements in the HBOT group (Fig. 5). These findings align with previous research evaluating the effects of HBOT on other FMS populations, where similar improvements in PFC and temporal brain area metabolism were observed when compared to control groups^20,24^. The PFC and temporal lobe, along with their interconnections, play pivotal roles in emotion regulation processes^39^. The PFC modulates emotional experiences and behaviors through its influence on temporal limbic areas, including the amygdala and insula, which ultimately impact well-being^40^. Furthermore, the PFC encompasses components of the pain perception system, particularly associated with aspects of emotion regulation, cognition, and attention related to pain perception^41^. Thus, it is conceivable that metabolic changes in these brain areas contributed to the significant improvements in emotional and physical symptoms observed after HBOT.

Beyond improvements in pain-related symptoms, the enhanced emotional state observed post-HBOT—characterized by reduced PTSD symptoms and depression rates and intensity—may further enhance daily functioning. Those results are in line with previous studies with veterans suffering from combat related PTSD^31–33^ as well as the previous results from study done on FMS related CSA^20,42^. In longitudinal evaluation of these veterans with combat-related PTSD, it was demonstrated that the beneficial effects of HBOT persisted even two years after treatment^31^. A longitudinal study is currently underway to determine whether the neuroplasticity and quality of life improvements observed in the current population of CSA survivors will also endure.

Pain, a pivotal element in FMS, was assessed through symptom questionnaires and laboratory evaluations of PPT and CPM. HBOT exhibited marginal significance improvement in pain measures associated with pressure and temperature (PPT and PPT-W) when compared to conventional medication. Nevertheless, clinically discernible improvements were noted in participants' subjective pain perception following HBOT compared to MED, alongside reported enhancements in functionality capacity. These observations suggest that the impact of HBOT may predominantly manifest in altering pain perception and its ramifications on daily function, in higher extent than affecting specific physical dimensions of pain. This supposition finds potential support in neuroimaging data revealing brain metabolic alterations in PFC regions involved in pain perception and emotional regulation neural pathways, as mentioned above.

Furthermore, HBOT versus MED yielded no significant different effects in CPM test. These outcomes diverge from those of a comparable study by Ablin et al.^24^, where a similar comparison between HBOT and MED demonstrated a significant group-by-time interaction favoring the HBOT group. However, Ablin's study cohort comprised individuals with FMS subsequent to TBI. The disparate findings between studies suggest potentially distinct neurological and physiological mechanisms underlying FMS etiologies. Further studies are required to comprehensively explore the distinctions between FMS etiologies, clinical presentation and treatment benefits.

As detailed in the results, HBOT was generally well-tolerated and safe in our study. The most common side effect observed in the HBOT group was mild middle ear barotrauma, which occurred in 29% of participants. It is noteworthy that these cases typically resolved spontaneously after discontinuing treatment for a few days. An additional phenomenon, previously reported in the literature^34^, should be recognized by all medical teams treating patients with HBOT. This phenomenon involves the surfacing of previously inaccessible memories from childhood and was reported by 8% of patients in our study. When these memories resurfaced, they occasionally came with emotional distress. However, it is essential to note that this emotional distress was generally relieved during the course of HBOT, and appropriate support was provided to address any psychological concerns. In contrast, the medication group exhibited a different profile of side effects. The most common side effects reported in this group included increased fatigue (37%), increased pain (29%), emotional distress (25%), and digestive/stomach pain (20%).

This study has several limitations. Firstly, there is a lack of long-term assessment of the effects of HBOT, which is currently being addressed through a 1-year longitudinal evaluation with participants who completed the treatment. Secondly, the study did not employ a dose–response protocol for HBOT. The HBOT protocol used, consisting of 60 daily sessions, 5 days per week, with 90 min of 100% oxygen at 2 ATA with air breaks every 20 min, was determined based on past clinical experience and research on HBOT effects in CSA, PTSD and other brain-related injuries. Future research could explore and compare different protocols and continuously monitor biomarkers to determine the most effective treatment protocol on an individual basis, considering the heterogeneity within the FMS patient population. Additionally, investigating a larger sample size will be essential in identifying the patients who stand to benefit the most from this treatment.

Another limitation of our study related to the prevalence of medication administration. The clinical decision-making process regarding medication selection for each participant encompassed the pain profile exhibited by each participant, alongside emotional and therapeutic pharmacological regimen, extending beyond FMS (as evidenced in Table 1). Given the prevalence of various psychiatric medications, some of which may serve as contraindications to Duloxetine, and with the primary objective of tailoring treatment specifically for FMS rather than other comorbidities that participants may contend with and receive pharmacological intervention for, the preference of the physician while discussion the different options with the patients was to administer Pregabalin in most cases within the current study cohort.

In conclusion, HBOT demonstrates the potential to improve both emotional and physical symptoms, as well as daily functioning in individuals with CSA-related FMS. Relief in pain may originate from alterations in pain perception and emotion regulation neural pathways, more than from changes in physical pain sensation. The magnitude of improvement with HBOT significantly exceeds that achieved through current recommended medications. These clinical improvements are closely correlated with the enhancement in brain perfusion and metabolism induced by HBOT. This study underscores the potential of HBOT as an effective therapeutic intervention for FMS patients.

## Methods

### Participants

The study included young adult females (age 18–45) with a history of CSA and a FMS diagnosis. Diagnosis of FMS was verified by a certified rheumatologist, and according to the updated 2016 diagnostic criteria^35^. Exclusion criteria included a history of traumatic brain injury (TBI), participants treated with Duloxetine (Cymbalta) or Pregabalin (Lyrica) in the past year, or any contraindications for this pharmaceutical treatment; Participants suffering from major psychiatric disorder such as schizophrenia, bi-polar disorder, or with suicidal attempt/s in the previous year. Other exclusion criteria were: HBOT for any other reason prior to their inclusion; Chest pathology (including active asthma); Inner ear disease; Claustrophobia; Inability to perform awake brain MRI test; Chronic renal failure (eGFR < 60 ml/min); Previous neurological conditions (e.g. Epilepsy, neuromuscular diseases, metabolic diseases, brain tumors etc.); Inability to sign informed consent. Smoking was not allowed during the HBOT period. Participants had to be engaged in psychotherapy on a weekly basis in the community.

### Trial design

A prospective, randomized, two active arms trial was conducted in the Sagol center for hyperbaric medicine and research, Shamir medical center, between the years 2020–2023. Following signing an informed consent, participants performed baseline evaluation, including brain imaging, questionnaires, pain tests as well as blood work and cognitive tests. Participants were then randomized to either HBOT or MED intervention in a 1:1 ratio using computerized randomization table, supervised by a blinded researcher. Post intervention evaluations were performed after end of HBOT sessions or after 3 months in the MED protocol. Intention to treat analysis was performed on all included participants who completed evaluations.

The study was approved by the institutional review board (IRB) of Shamir medical center (No. 0008-20-ASF), all participants signed informed consent prior to inclusion. All methods were performed in accordance with relevant guidelines and regulations. The study was registered with Clinicaltrials.gov, number NCT04316702 posted on 20/03/2020.

### Procedure

HBOT protocol was administered in a multi-place chamber and included total of 60 daily HBOT sessions, five days per week. Each session included inhalation of 100% oxygen by mask at 2 ATA for 90 min, with five minutes' air breaks every 20 min.

In the MED protocol, participants were assigned for pharmacological treatment with one of the two medications FDA approved and currently licensed for the treatment of FMS by the FDA and in Israel, i.e. Duloxetine or Pregabalin, based on clinical judgment. Treatment with Pregabalin started at a dose of 75 mg at bedtime while treatment with Duloxetine started at a dose of 30 mg a day in the morning. Every 2 weeks, participants were evaluated, and doses were adjusted as necessary and tolerated. The importance of engaging in physical exercise in FMS treatment, according to Israeli guidelines of treatment, was explained and recommended to all participants.

The research team elucidated the advantages of both interventions and made efforts to maintain parity in tracking and monitoring across both cohorts. Participants from both groups were required to be engaged in psychotherapy sessions on a weekly basis (minimum), with a well-trained mental health professional in their community, during the treatment period. In addition, participants from both groups were asked to write a daily journal expressing their thoughts and emotions arising in the treatment, this information was a foundation for a correspondence (written questions and answers) with an assigned accompanying research team member (mental health professionals), for further support in the therapeutic process.

### Primary end point

Primary end point was the score of FMS impact questionnaire (FIQ, Hebrew version)^43^, an assessment and evaluation instrument developed to measure FMS patient’s status, progress and outcomes. It has been designed to measure the components of health status that are believed to be most affected by FMS. Score ranges from 0 to 100, higher score indicates a greater impact of the syndrome on the person. Test–retest reliability of the Hebrew version was found high (r = 0.96 for physical functioning, and 0.80–0.96 for other items of FIQ^43^).

### Secondary end points

#### Questionnaires

Self-report questionnaires evaluating physical, emotional state and quality of life: Wide Spread pain index (WPI) and Symptom severity score (SSS)^44^ for FMS diagnostics^35^; Brief symptom inventory—18 (BSI-18)^45^; Beck depression inventory (BDI-II)^46^; PTSD symptom scale interview (PSS-I)^47^; Medical somatic dissociation questionnaire (MSDQ)^48^; The RAND health status survey, short form-36 (SF-36) questionnaire^49,50^; Childhood trauma questionnaire (CTQ), a screening tool for histories of abuse and neglect^51^, was used at baseline evaluation for the assessment of childhood trauma. Further explanations of each questionnaire and its measures, as well as reliability data, are detailed in Supplementary Material S4.

### Pain test

Evaluation of pain profile included pressure pain threshold (PPT) by applying pressure to the upper trapezius muscle using handheld algometer with a circular 1 cm^2^ probe (AlgoMed, Medoc LTD, Israel)^52^. PPTs were measured three times at the upper trapezius muscle. The baseline pressure applied was 0 kPa, with incremental increases of 30 kPa per second, up to a maximal pressure of 1000 kPa. The participant was instructed to report when the sensation changed from pressure to pain, at which point the probe was removed. The average of the second and third measurements was used in further analyses.

For evaluating conditioned pain modulation (CPM), The PPT test was repeated during immersion of the non-dominant hand in 10 °C cold water (PPT-W). CPM was evaluated by computing the difference in mean pain intensity induced by the pressure stimulus before and during the cold water immersion of the opposite hand. Thus, effective pain inhibitory mechanisms were represented by higher (positive) values^53^.

### Brain perfusion SPECT imaging

Brain single photon emission computed tomography (SPECT) was conducted with 925–1110 MBq (25–30 mCi) of technetium-99m-Ethyl-cysteinate-dimmer (Tc-99m-ECD) at 40–60 min post injection, using a dual detector gamma camera (ECAM or Symbia T, Siemens Medical Systems) equipped with high resolution collimators. Data was acquired in 3-degree steps and reconstructed iteratively by the Chang method (µ = 0.12/cm) attenuation correction. Pre- and post-treatment SPECT were normalized to maximal whole brain activity. SPECT images were reoriented into Talairach space using NeuroGam (Segami Corporation, Culombia, MS, USA) for identification of Brodmann cortical areas, and to compute the mean perfusion in each BA.

Other post intervention endpoints included in this study were magnetic resonance imaging (MRI) and functional magnetic resonance imaging (fMRI), blood work, self-image and assault drawings, and cognitive evaluations. Results are to be analyzed and included in future publications.

### Statistical analysis

Continuous data are expressed as means ± standard deviations (SD). Two-tailed independent t-tests were performed to compare variables between groups when a normality assumption held according to a Kolmogorov–Smirnov test. Net effect sizes were evaluated using Cohen’s d method, defined as the improvement from baseline after MED intervention, subtracted from the improvement after HBOT, divided by the pooled standard deviation of the composite score. Categorical data were expressed in numbers and percentages, compared by chi-square/Fisher’s exact tests. To evaluate HBOT’s vs MED effect, a repeated-measure ANOVA mixed-model was used to compare post-treatment and pre-treatment data. The model included group-by-time interactions. In questionnaire analysis, a Bonferroni correction was used for multiple comparisons. In SPECT analysis, for each BA activation was normalized to the group median level. FDR correction was used for multiple comparisons. A value of p < 0.05 was considered significant. Pearson’s correlations were performed between scores or SPECT activation changes and the change in questionnaire scores and pain test before and after treatment. Data analysis was performed using MATLAB R2021b (MathWorks, Natick, MA) Statistics and Machine Learning Toolbox.

### Sample size calculation

In light of previous findings, estimated sample size was calculated based on a reduction of 30 points in the HBOT group and a reduction of 10 points in the medication group, in the primary endpoint measure, with a standard deviation of 24^20,54^. Assuming a power of 80%, and 5% two-sided level of significance, a total of 46 participants would be required, 23 participants in each group. Considering a dropout rate of 20%, the total sample size required is 58. The actual enrolment was 60 informed consents, with dropout rate of 20% as estimated.

### Supplementary Information

Supplementary Information.

## Data Availability

The datasets used and analyzed during the current study are available from the corresponding author on reasonable request.

## References

[1] Schmidt-Wilcke T, Clauw DJ (2011). Fibromyalgia: From pathophysiology to therapy. Nat. Rev. Rheumatol..

[2] Buskila D (2009). Developments in the scientific and clinical understanding of fibromyalgia. Arthritis Res. Ther..

[3] Yunus MB (2007). Role of central sensitization in symptoms beyond muscle pain, and the evaluation of a patient with widespread pain. Best Pract. Res. Clin. Rheumatol..

[4] Ablin JN, Efrati S, Buskila D (2016). Building up the pressure on chronic pain. Clin. Exp. Rheumatol..

[5] Walker E (1997). Psychosocial factors in fibromyalgia compared with rheumatoid arthritis: II. Sexual, physical, and emotional abuse and neglect. Psychosom. Med..

[6] Maugars Y, Berthelot JM, Le Goff B, Darrieutort-Laffite C (2021). Fibromyalgia and associated disorders: From pain to chronic suffering, from subjective hypersensitivity to hypersensitivity syndrome. Front. Med. (Lausanne).

[7] Gunnar, M. R. & Vazquez, D. Stress neurobiology and developmental psychopathology. *Dev. Psychopathol. Vol. Two Dev. Neurosci.* 533–577 (2015).

[8] Bremner JD (2003). MRI and PET study of deficits in hippocampal structure and function in women with childhood sexual abuse and posttraumatic stress disorder. Am. J. Psychiatry.

[9] Liberzon I, Sripada CS (2008). The functional neuroanatomy of PTSD: A critical review. Progr. Brain Res..

[10] Blanco L (2015). Neurological changes in brain structure and functions among individuals with a history of childhood sexual abuse: A review. Neurosci. Biobehav. Rev..

[11] McCrory E, De Brito SA, Viding E (2011). The impact of childhood maltreatment: A review of neurobiological and genetic factors. Front. Psychiatry.

[12] Schnurr PP, Green BL (2004). Understanding relationships among trauma, post-tramatic stress disorder, and health outcomes. Adv. Mind-Body Med..

[13] Sherin JE, Nemeroff CB (2011). Post-traumatic stress disorder: The neurobiological impact of psychological trauma. Dialog. Clin. Neurosci..

[14] Macfarlane GJ (2017). EULAR revised recommendations for the management of fibromyalgia. Ann. Rheum. Dis..

[15] Shor DB-A (2017). Adherence and persistence with drug therapy among fibromyalgia patients: Data from a large health maintenance organization. J. Rheumatol..

[16] Hauser W, Walitt B, Fitzcharles MA, Sommer C (2014). Review of pharmacological therapies in fibromyalgia syndrome. Arthritis Res. Ther..

[17] Hadanny A, Efrati S (2020). The hyperoxic–hypoxic paradox. Biomolecules.

[18] Efrati S, Ben-Jacob E (2014). Reflections on the neurotherapeutic effects of hyperbaric oxygen. Expert. Rev. Neurother..

[19] Gottfried I, Schottlender N, Ashery U (2021). Hyperbaric oxygen treatment-from mechanisms to cognitive improvement. Biomolecules..

[20] Hadanny A (2018). Hyperbaric oxygen therapy can induce neuroplasticity and significant clinical improvement in patients suffering from fibromyalgia with a history of childhood sexual abuse-randomized controlled trial. Front. Psychol..

[21] Efrati S (2015). Hyperbaric oxygen therapy can diminish fibromyalgia syndrome—Prospective clinical trial. PLoS One.

[22] Efrati S (2013). Hyperbaric oxygen induces late neuroplasticity in post stroke patients—randomized, prospective trial. PLoS One.

[23] Hadanny A (2020). Cognitive enhancement of healthy older adults using hyperbaric oxygen: A randomized controlled trial. Aging (Albany NY).

[24] Ablin JN (2023). Hyperbaric oxygen therapy compared to pharmacological intervention in Fibromyalgia patients following traumatic brain injury: A randomized, controlled trial. PLoS One.

[25] Tal S (2015). Hyperbaric oxygen may induce angiogenesis in patients suffering from prolonged post-concussion syndrome due to traumatic brain injury. Restor. Neurol. Neurosci..

[26] Boussi-Gross R (2015). Improvement of memory impairments in poststroke patients by hyperbaric oxygen therapy. Neuropsychology.

[27] Boussi-Gross R (2013). Hyperbaric oxygen therapy can improve post concussion syndrome years after mild traumatic brain injury—Randomized prospective trial. PLoS One.

[28] Hadanny A (2015). Hyperbaric oxygen can induce neuroplasticity and improve cognitive functions of patients suffering from anoxic brain damage. Restor. Neurol. Neurosci..

[29] Yildiz S (2004). A new treatment modality for fibromyalgia syndrome: Hyperbaric oxygen therapy. J. Int. Med. Res..

[30] Zilberman-Itskovich S (2022). Hyperbaric oxygen therapy improves neurocognitive functions and symptoms of post-COVID condition: Randomized controlled trial. Sci. Rep..

[31] Doenyas-Barak K (2022). Hyperbaric oxygen therapy for veterans with treatment-resistant PTSD: A longitudinal follow-up study. Mil. Med..

[32] Doenyas-Barak K (2022). Hyperbaric oxygen therapy improves symptoms, brain's microstructure and functionality in veterans with treatment resistant post-traumatic stress disorder: A prospective, randomized, controlled trial. PLoS One.

[33] Keren Doenyas-Barak IK, Erez L, Rachel M, Rachel LW, Rahav B-G, Shai E (2023). The use of hyperbaric oxygen for veterans with PTSD: Basic physiology and current available clinical data. Front. Neurosci..

[34] Efrati S (2018). Recovery of repressed memories in fibromyalgia patients treated with hyperbaric oxygen—Case series presentation and suggested bio-psycho-social mechanism. Front. Psychol..

[35] Wolfe F (2016). 2016 Revisions to the 2010/2011 fibromyalgia diagnostic criteria. Semin. Arthritis Rheum..

[36] Yehuda R (2015). Post-traumatic stress disorder. Nat. Rev. Dis. Primers.

[37] Mueller SG (2015). Evidence for disrupted gray matter structural connectivity in posttraumatic stress disorder. Psychiatry Res. Neuroimaging.

[38] Teicher MH, Samson JA, Anderson CM, Ohashi K (2016). The effects of childhood maltreatment on brain structure, function and connectivity. Nat. Rev. Neurosci..

[39] Dixon ML, Thiruchselvam R, Todd R, Christoff K (2017). Emotion and the prefrontal cortex: An integrative review. Psychol. Bull..

[40] Gross JJ, John OP (2003). Individual differences in two emotion regulation processes: Implications for affect, relationships, and well-being. J. Personal. Soc. Psychol..

[41] Mercer Lindsay N, Chen C, Gilam G, Mackey S, Scherrer G (2021). Brain circuits for pain and its treatment. Sci. Transl. Med..

[42] Lev-Wiesel R, Bechor Y, Daphna-Tekoah S, Hadanny A, Efrati S (2018). Brain and mind integration: Childhood sexual abuse survivors experiencing hyperbaric oxygen treatment and psychotherapy concurrently. Front. Psychol..

[43] Buskila D, Neumann L (1996). Assessing functional disability and health status of women with fibromyalgia: Validation of a Hebrew version of the Fibromyalgia Impact Questionnaire. J. Rheumatol..

[44] Wolfe F, Häuser W (2011). Fibromyalgia diagnosis and diagnostic criteria. Ann. Med..

[45] Canetti L, Shalev AY, De-Nour AK (1994). Israeli adolescents' norms of the Brief Symptom Inventory (BSI). Isr. J. Psychiatry Relat. Sci..

[46] Beck, A. T., Steer, R. A. & Brown, G. Beck depression inventory–II. *Psychol. Assess.* (1996).

[47] Foa EB, Tolin DF (2000). Comparison of the PTSD symptom scale-interview version and the clinician-administered PTSD scale. J. Trauma Stress..

[48] Daphna-Tekoah S, Lev-Wiesel R, Israeli D, Balla U (2019). A novel screening tool for assessing child abuse: The medical somatic dissociation questionnaire—MSDQ. J. Child Sex. Abuse.

[49] McHorney CA, Ware JE, Lu JF, Sherbourne CD (1994). The MOS 36-item Short-Form Health Survey (SF-36): III. Tests of data quality, scaling assumptions, and reliability across diverse patient groups. Med. Care.

[50] McHorney CA, Ware JE, Raczek AE (1993). The MOS 36-Item Short-Form Health Survey (SF-36): II. Psychometric and clinical tests of validity in measuring physical and mental health constructs. Med. Care.

[51] Fink LA, Bernstein D, Handelsman L, Foote J, Lovejoy M (1995). Initial reliability and validity of the childhood trauma interview: A new multidimensional measure of childhood interpersonal trauma. Am. J. Psychiatry.

[52] Alsouhibani A, Vaegter HB, Hoeger Bement M (2018). Systemic exercise-induced hypoalgesia following isometric exercise reduces conditioned pain modulation. Pain Med..

[53] Chalaye P, Devoize L, Lafrenaye S, Dallel R, Marchand S (2013). Cardiovascular influences on conditioned pain modulation. PAIN®.

[54] Arnold LM (2008). A 14-week, randomized, double-blinded, placebo-controlled monotherapy trial of pregabalin in patients with fibromyalgia. J. Pain.

