# Supplementary material for: Hyperbaric oxygen therapy vs. pharmacological intervention in adults with fibromyalgia related to childhood sexual abuse: prospective, randomized clinical trial
Source: Sci Rep. 2024 May 21;14:11599. doi: 10.1038/s41598-024-62161-5 (PMC11109175; doi:10.1038/s41598-024-62161-5)
Supplement: Supplementary file 1 — Supplementary Information. [file 41598_2024_62161_MOESM1_ESM.pdf]

Hyperbaric Oxygen Therapy vs. Pharmacological Intervention in Adults with Fibromyalgia Related to Childhood Sexual Abuse: Prospective, Randomized Clinical Trial

Boussi-Gross Rahav[1], Catalogna Merav[1], Lang Erez[1,2], Shamai Zipora [1], Ablin Jacob N. [6,2], Aloush Valerie [6,2], Doenyas-Barak Keren [1,2,3], Lorberboym Mordechai[4], Lev-Wiesel Rachel[1,5], Efrati Shai[1,2,3]

[1]Sagol Center for Hyperbaric Medicine and Research, Shamir (Assaf Harofeh) Medical Center, Zerifin, Israel.

[2]School of Medicine, Tel- Aviv University, Tel-Aviv, Israel.

[3]Sagol School of Neuroscience, Tel-Aviv University, Tel-Aviv, Israel.

[4]Nuclear Medicine Institute, Shamir (Assaf Harofeh) Medical Center, Zerifin, Israel.

[5]The Emili Sagol CAT Research Center, Graduate School of Creative Arts Therapies, University of Haifa, Israel

[6]Tel Aviv Sourasky Medical Center, Israel

Supplementary Table S1: Complete questionnaires analysis.

	HBOT Group				MED Group				ANOVA**			
	**PRE**	**POST**	**Change**	**P-value**	**PRE**	**POST**	**Change**	**P-value**	**P-value Baseline**	**Effect size***	**F**	**P-value**
**N**	24				24							
**Wide spread pain index WPI**	13.9±4.1	9.4±6.0	-4.5±5.9	0.001	13.1±3.9	13.4±2.9	0.3±3.9	0.717	0.503	-0.971	11.322	**0.002**
**Symptom severity score SSS**	10.3±1.6	8.0±2.5	-2.3±2.8	0.001	10.5±1.1	10.4±1.2	-0.1±1.4	0.671	0.681	-0.988	11.706	**0.001**
**Fibromyalgia impact questionnaire-revised (FIQ-R)**												
Total FIQ-R	67.5±13.4	43.2±20.0	-24.3±20.9	0.000	69.2±13.6	66.3±12.8	-2.9±11.1	0.207	0.665	-1.277	19.562	**<0.000**
Function	18.0±5.9	11.7±6.2	-6.4±6.4	0.000	19.6±5.9	19.1±5.6	-0.5±4.7	0.631	0.386	-1.050	13.239	**0.001**
Overall impact	13.8±4.5	6.7±5.7	-7.1±6.9	0.000	12.8±4.3	11.5±4.0	-1.2±3.9	0.129	0.446	-1.043	13.058	**0.001**
Symptoms	35.6±6.5	24.8±10.4	-10.8±11.4	0.000	36.9±5.7	35.6±5.6	-1.2±4.8	0.231	0.506	-1.100	14.525	**<0.000**
**Brief symptoms inventory – BSI**												
Total BSI	45.3±13.4	32.1±19.1	-13.2±21.6	0.006	46.2±9.9	44.7±9.2	-1.5±11.4	0.514	0.793	-0.677	5.499	0.023
Somatization	15.3±4.8	10.1±6.6	-5.2±7.4	0.002	14.5±4.5	13.8±3.9	-0.7±4.4	0.441	0.547	-0.737	6.518	0.014
Depression	14.0±5.9	10.5±6.7	-3.5±7.7	0.035	15.7±4.5	14.7±4.7	-1.0±4.4	0.279	0.299	-0.404	1.959	0.168
Anxiety	16.0±5.4	11.5±7.4	-4.5±8.2	0.013	16.1±3.3	16.3±3.4	0.2±4.4	0.855	0.925	-0.705	5.966	0.018
**PTSD symptom scale– PSS**												
PTSD Severity	35.2±9.1	25.4±12.8	-9.8±12.6	0.001	36.0±7.1	35.1±8.9	-0.9±8.7	0.610	0.743	-0.824	8.150	0.006
Re-experiencing	9.6±3.4	7.0±4.4	-2.6±3.7	0.002	9.3±3.7	9.6±3.6	0.3±3.0	0.592	0.781	-0.870	9.080	0.004
Avoidance	14.7±4.5	10.7±5.6	-4.0±5.7	0.002	15.1±3.4	14.4±4.3	-0.7±4.8	0.503	0.752	-0.638	4.881	0.032
Arousal	11.0±3.3	7.8±4.2	-3.2±5.1	0.006	11.7±2.8	11.1±3.0	-0.6±3.0	0.345	0.436	-0.627	4.720	0.035
**BECK depression inventory**	33.8±11.2	24.2±15.3	-9.6±15.4	0.006	35.3±12.0	35.6±9.3	0.3±10.9	0.883	0.662	-0.744	6.638	0.013
**Medical somatic dissociation questionnaire MSDQ**												
Total MSDQ	2.1±0.6	1.5±0.7	-0.6±0.8	0.001	2.2±0.7	2.2±0.7	0.0±0.4	0.704	0.780	-1.014	12.336	**0.001**
Somatization	2.8±0.7	1.9±1.0	-0.9±1.1	0.001	2.5±0.7	2.5±0.8	-0.0±0.6	0.827	0.327	-0.917	10.083	0.003
Depression symptoms	2.5±0.8	1.8±0.9	-0.7±1.0	0.002	2.8±0.8	2.7±0.8	-0.0±0.6	0.789	0.302	-0.825	8.162	0.006
Dissociative manifestations	1.7±0.9	1.1±0.8	-0.6±1.0	0.008	1.7±0.9	1.8±0.9	0.1±0.7	0.586	0.978	-0.784	7.378	**0.009**
**RAND health status survey short form 36 (SF-36)**												

Physical functioning	37.9±18.3	57.1±20.5	19.2±17.4	0.000	42.7±21.3	41.9±19.6	-0.8±14.1	0.775	0.416	1.261	19.089	**<0.000**
Physical limitations	8.3±17.2	43.8±35.5	35.4±36.1	0.000	10.4±19.0	12.5±21.7	2.1±31.2	0.747	0.698	0.989	11.729	0.001
Emotional limitations	13.9±27.1	44.4±41.6	30.6±38.0	0.001	6.9±13.5	18.1±25.4	11.1±28.9	0.073	0.277	0.576	3.982	0.052
Energy	14.4±11.4	41.0±22.2	26.7±21.2	0.000	15.0±12.3	18.8±17.3	3.8±17.8	0.312	0.859	1.170	16.429	**0.000**
Emotional wellbeing	39.0±16.1	54.2±19.8	15.2±22.7	0.003	36.5±18.8	37.8±14.0	1.3±17.9	0.719	0.630	0.676	5.477	0.024
Social function	28.6±22.1	53.6±20.9	25.0±27.1	0.000	35.9±18.9	38.0±23.5	2.1±22.3	0.652	0.235	0.923	10.232	0.003
Pain Domain	19.9±19.5	45.1±25.5	25.2±19.5	0.000	21.1±19.1	22.2±19.9	1.0±20.2	0.803	0.827	1.215	17.715	**<0.000**
General Health Domain	29.0±16.6	45.6±21.8	16.7±21.2	0.001	32.1±17.6	33.3±18.9	1.2±18.4	0.743	0.539	0.776	7.230	0.010
Physical component score	23.8±12.4	47.9±21.5	24.1±17.9	0.000	26.6±14.0	27.5±12.6	0.9±13.6	0.7521	0.475	1.464	25.730	**<0.000**
Mental component score	24.0±13.1	48.3±21.5	24.3±20.8	0.000	23.6±10.5	28.2±14.5	4.6±17.1	0.2026	0.913	1.039	12.966	**0.001**

Data are presented as mean ± SD; Bold, significant after Bonferroni correction; * Cohen's d net effect size; ** group-by-time interaction

Supplementary Table S2: Pain test analysis.

		HBOT Group				Medication Group				ANOVA Group-by-Time Interaction			
		**PRE**	**POST**	**Change**	**P-value**	**PRE**	**POST**	**Change**	**P-value**	**P-value Baseline**	**Net effect size***	**F**	**P-value**
N		22				24							
Pain Test	PPT (KPa)	291.9±113.1	362.8±144.0	70.8±90.6	0.001	259.4±127.0	279.4±118.4	20.1±85.7	0.263	0.376	0.57	3.81	0.057
	PPT-W (KPa)	390.5±156.2	464.1±146.4	73.6±121.4	0.010	350.4±176.2	354.7±151.1	4.4±121.2	0.861	0.430	0.57	3.73	0.060
	CPM (KPa)	98.6±81.9	101.3±84.5	2.8±116.9	0.913	91.0±70.2	75.3±80.7	-15.7±99.4	0.447	0.742	0.17	0.33	0.566

* Cohen’s d net effect size. Abbreviations: PPT, Pressure threshold; PPT-W, Pressure threshold in water; CPM, Conditioned pain modulation.

Supplementary Table S3: SPECT data. Complete Brodmann areas analysis.

					ANOVA**	
	**HBOT group P-value**	**Med group P-value**	**Baseline comparison p-value**	**Net effect size***	**F**	**P-value**
BA 4 LEFT	0.393	0.986	0.420	-0.180	0.390	0.535
BA 4 RIGHT	0.521	0.327	0.477	0.337	1.362	0.249
BA 5 LEFT	0.857	0.307	0.521	-0.156	0.293	0.591
BA 5 RIGHT	0.601	0.778	0.512	-0.163	0.317	0.576
BA 6 LEFT	**0.006**	0.545	0.084	0.731	6.420	**0.015**
BA 6 RIGHT	0.100	0.682	0.449	0.439	2.317	0.135
BA 7 LEFT	0.998	0.791	0.988	0.058	0.040	0.841
BA 7 RIGHT	0.997	0.433	0.719	-0.172	0.356	0.554
BA 8 LEFT	0.167	0.952	0.793	0.234	0.656	0.422
BA 8 RIGHT	0.177	0.490	0.873	0.443	2.358	0.131
BA 9 LEFT	0.329	0.654	0.386	-0.286	0.980	0.327
BA 9 RIGHT	0.095	0.200	0.937	0.629	4.747	**0.035**
BA 10 LEFT	0.911	0.498	0.805	0.129	0.201	0.656
BA 10 RIGHT	0.150	0.353	0.415	0.504	3.054	0.087
BA 11 LEFT	0.198	0.823	0.930	0.304	1.109	0.298
BA 11 RIGHT	0.523	0.109	0.684	0.493	2.921	0.094
BA 17 LEFT	0.118	0.567	0.753	0.413	2.051	0.159
BA 17 RIGHT	0.582	**0.000**	0.092	0.753	6.803	**0.012**
BA 18 LEFT	0.448	0.505	0.930	-0.011	0.002	0.969
BA 18 RIGHT	0.629	0.073	0.764	0.430	2.223	0.143
BA 19 LEFT	0.065	0.903	0.275	0.351	1.480	0.230
BA 19 RIGHT	**0.049**	0.881	0.427	0.405	1.969	0.167
BA 20 LEFT	0.270	0.547	0.858	0.355	1.512	0.225
BA 20 RIGHT	0.131	0.789	0.820	0.382	1.754	0.192
BA 21 LEFT	0.289	0.386	0.905	-0.040	0.020	0.889
BA 21 RIGHT	0.872	0.974	0.477	-0.036	0.016	0.900
BA 22 LEFT	0.855	0.870	0.232	-0.071	0.061	0.806
BA 22 RIGHT	0.299	0.828	0.553	0.142	0.242	0.625

BA 23 LEFT	0.136	**0.010**	0.650	-0.434	2.257	0.140
BA 23 RIGHT	0.846	**0.020**	0.531	-0.519	3.228	0.079
BA 24 LEFT	0.209	0.181	0.699	0.070	0.058	0.810
BA 24 RIGHT	0.673	0.747	0.640	-0.011	0.001	0.969
BA 25 LEFT	0.983	0.429	0.794	0.195	0.454	0.504
BA 25 RIGHT	0.756	0.620	0.731	0.167	0.336	0.565
BA 28 LEFT	0.063	0.324	0.662	0.584	4.095	**0.049**
BA 28 RIGHT	0.966	0.489	0.669	-0.147	0.261	0.612
BA 31 LEFT	0.543	0.396	0.606	-0.110	0.146	0.704
BA 31 RIGHT	0.542	0.759	0.863	-0.180	0.387	0.537
BA 32 LEFT	0.161	**0.048**	0.644	0.724	6.285	**0.016**
BA 32 RIGHT	0.107	0.353	0.613	0.527	3.333	0.074
BA 36 LEFT	**0.004**	0.249	0.144	0.949	10.797	**0.002**
BA 36 RIGHT	0.979	0.619	0.669	-0.107	0.138	0.712
BA 37 LEFT	0.518	0.702	0.631	-0.029	0.010	0.921
BA 37 RIGHT	0.110	0.880	0.467	0.291	1.019	0.318
BA 38 LEFT	0.271	0.129	0.998	0.548	3.609	0.064
BA 38 RIGHT	0.802	0.741	0.625	-0.011	0.001	0.971
BA 39 LEFT	0.935	0.639	0.589	-0.092	0.101	0.752
BA 39 RIGHT	0.108	0.355	0.704	0.153	0.281	0.598
BA 40 LEFT	0.830	0.215	0.294	-0.313	1.174	0.284
BA 40 RIGHT	**0.016**	0.531	0.189	0.653	5.109	**0.029**
BA 44 LEFT	0.251	0.273	0.750	0.465	2.599	0.114
BA 44 RIGHT	0.689	0.084	0.872	0.469	2.645	0.111
BA 45 LEFT	0.285	0.602	0.580	0.102	0.125	0.725
BA 45 RIGHT	0.523	0.238	0.972	0.383	1.756	0.192
BA 46 LEFT	**0.033**	0.238	0.444	0.727	6.340	**0.015**
BA 46 RIGHT	0.935	0.290	0.697	0.262	0.826	0.368
BA 47 LEFT	0.496	0.550	0.690	0.264	0.834	0.366
BA 47 RIGHT	0.460	0.895	0.501	0.124	0.185	0.669

* Cohen's d net effect size;** Group-by-time interaction; BA, Brodmann area

Supplementary Material S4: Questionnaires

Self-report questionnaires evaluating physical, emotional state and quality of life:

Wide spread pain index (WPI) and **symptom severity score** (SSS) [1] - WPI is a 0–19 count of the number of body regions reported as painful by the patient. SSS assesses on a 0–3 severity scale a series of symptoms that are characteristic of fibromyalgia: fatigue, non-refreshed sleep, cognitive problems, and the extent of somatic symptom reporting. The items are combined into a 0–12 Symptom Severity (SS) scale. Fibromyalgia diagnostic criteria are expressed as: WPI >= 7 and SSS >= 5, or WPI = 3-6, and SSS >= 9 [2].

The Brief symptom inventory – 18 (BSI-18) [3] was used to evaluate psychological distress, generating a summary scale, and three subscales: depression, anxiety, and somatization. Test-retest reliability was reported from r=0.68 to 0.84 in subscales and 0.90 for summary scale [4].

Beck depression inventory (BDI-II) [5] is a 21-question multiple choice self-report inventory, designed to measure severity of depression. The questions refer to the subject's feelings in the past week, and has four possible answers for each question, signifying increased intensity. a total score is compared to a key to determine depression's severity: 0-13 indicates minimal depression, 14-19 mild, 20-28 moderate, and 29-63 indicates severe depression. Test-retest reliability was reported high (r=0.96) [6].

The PTSD symptom scale interview (PSS-I) [7] was used to assess the presence and severity of DSM-IV PTSD symptoms. PTSD severity is determined by totaling the 17 PSS-I symptom ratings, scores range from 0-51. Test-retest reliability measures were found high in relevant populations (r=0.80 to 0.83) [8,9].

Medical somatic dissociation questionnaire (MSDQ) [10] was used to assess somatic dissociation. Composite scores are created into three factors: Physical, psychological and dissociative manifestation. MSDQ is a novel screening tool for assessing dissociation, it demonstrated powerful internal consistency, reliability and convergent validity [10], however test-retest reliability data were not yet reported.

The RAND health status survey, short form-36 (SF-36) questionnaire [11,12] a self-report measure that evaluates quality of life using several measures: physical functioning;

bodily pain; role limitations due to physical health problems; role limitations due to personal or emotional health; general mental health; social functioning; energy/fatigue; and general health perception. Physical component summary (PCS) and mental component summary (MCS) were calculated using these 8 measures (four in each component). Test-retest reliability of calculated subscales was found moderate to high (r=0.58 to 0.82) [13].

Childhood trauma questionnaire (CTQ), a screening tool for histories of abuse and neglect [14], was used at baseline evaluation for the assessment of childhood trauma. The self-report includes a 28-item test that measures 5 types of maltreatment – emotional, physical, and sexual abuse, and emotional and physical neglect. Each subscale score ranges from 5 (no history of abuse or neglect) to 25 (very extreme history of abuse and neglect).

References

1 Wolfe, F. & Häuser, W. Fibromyalgia diagnosis and diagnostic criteria. *Annals of medicine* **43**, 495-502 (2011).

2 Wolfe, F. *et al.* 2016 Revisions to the 2010/2011 fibromyalgia diagnostic criteria. *Semin Arthritis Rheum* **46**, 319-329, doi:10.1016/j.semarthrit.2016.08.012 (2016).

3 Canetti, L., Shalev, A. Y. & De-Nour, A. K. Israeli adolescents' norms of the Brief Symptom Inventory (BSI). *Isr J Psychiatry Relat Sci* **31**, 13-18 (1994).

4 Derogatis, L. R. *BSI 18, Brief Symptom Inventory 18: Administration, scoring and procedures manual*. (NCS Pearson, Incorporated, 2001).

5 Beck, A. T., Steer, R. A. & Brown, G. Beck depression inventory–II. *Psychological assessment* (1996).

6 Sprinkle, S. D. *et al.* Criterion validity, severity cut scores, and test-retest reliability of the Beck Depression Inventory-II in a university counseling center sample. *Journal of counseling psychology* **49**, 381 (2002).

7 Foa, E. B. & Tolin, D. F. Comparison of the PTSD Symptom Scale-Interview Version and the Clinician-Administered PTSD scale. *J Trauma Stress* **13**, 181-191, doi:10.1023/A:1007781909213 (2000).

8 Foa, E. B., Cashman, L., Jaycox, L. & Perry, K. The validation of a self-report measure of posttraumatic stress disorder: the Posttraumatic Diagnostic Scale. *Psychological assessment* **9**, 445 (1997).

9 Foa, E. B., Riggs, D. S., Dancu, C. V. & Rothbaum, B. O. Reliability and validity of a brief instrument for assessing post-traumatic stress disorder. *Journal of traumatic stress* **6**, 459-473 (1993).

10 Daphna-Tekoah, S., Lev-Wiesel, R., Israeli, D. & Balla, U. A novel screening tool for assessing child abuse: the medical somatic dissociation questionnaire–MSDQ. *Journal of child sexual abuse* **28**, 526-543 (2019).

11 McHorney, C. A., Ware, J. E., Jr., Lu, J. F. & Sherbourne, C. D. The MOS 36-item Short-Form Health Survey (SF-36): III. Tests of data quality, scaling assumptions, and reliability across diverse patient groups. *Med Care* **32**, 40-66 (1994).

12 McHorney, C. A., Ware, J. E., Jr. & Raczek, A. E. The MOS 36-Item Short-Form Health Survey (SF-36): II. Psychometric and clinical tests of validity in measuring physical and mental health constructs. *Med Care* **31**, 247-263 (1993).

13 VanderZee, K. I., Sanderman, R. & Heyink, J. A comparison of two multidimensional measures of health status: the Nottingham Health Profile and the RAND 36-Item Health Survey 1.0. *Quality of life research* **5**, 165-174 (1996).

14 Fink, L. A., Bernstein, D., Handelsman, L., Foote, J. & Lovejoy, M. Initial reliability and validity of the childhood trauma interview: a new multidimensional measure of childhood interpersonal trauma. *Am J Psychiatry* **152**, 1329-1335, doi:10.1176/ajp.152.9.1329 (1995).
